# High filamin-C expression predicts enhanced invasiveness and poor outcome in glioblastoma multiforme

**DOI:** 10.1038/s41416-019-0413-x

**Published:** 2019-03-14

**Authors:** Muhammad Kamil, Yoshinari Shinsato, Nayuta Higa, Takuro Hirano, Masashi Idogawa, Tomoko Takajo, Kentaro Minami, Michiko Shimokawa, Masatatsu Yamamoto, Kohichi Kawahara, Hajime Yonezawa, Hirofumi Hirano, Tatsuhiko Furukawa, Koji Yoshimoto, Kazunori Arita

**Affiliations:** 1 Department of Neurosurgery, Graduate School of Medical and Dental Sciences, Kagoshima University, Kagoshima, Japan; 2 Department of Molecular Oncology, Graduate School of Medical and Dental Sciences, Kagoshima University, Kagoshima, Japan; 3 Department of Neurosurgery, Faculty of Medicine, Airlangga University, Surabaya, Indonesia; 4 Department of Digestive Surgery, Breast and Thyroid Surgery, Graduate School of Medical and Dental Sciences, Kagoshima University, Kagoshima, Japan; 5 Department of Medical Genome Sciences, Research Institute for Frontier Medicine, Sapporo Medical University, Sapporo, Japan; 6 Center for the Research of Advanced Diagnosis and Therapy of Cancer, Graduate School of Medical and Dental Sciences, Kagoshima University, Kagoshima, Japan

**Keywords:** Prognostic markers, Actin, Cancer epidemiology, CNS cancer, Cell invasion

## Abstract

**Background:**

Glioblastoma multiforme (GBM), the most common brain malignancy in adults, is generally aggressive and incurable, even with multiple treatment modalities and agents. Filamins (FLNs) are a group of actin-binding proteins that regulate the actin cytoskeleton in cells. However, the role of FLNs in malignancies—particularly in GBM—is unclear.

**Methods:**

The relation between FLNC expression and overall survival in GBM was evaluated by the Kaplan−Meier analysis using GBM patients from the Kagoshima University Hospital (*n* = 90) and data from the Cancer Genome Atlas (TCGA) (*n* = 153). To assess FLNC function in GBM, cell migration and invasion were examined with Transwell and Matrigel invasion assays using FLNC-overexpressing U251MG and LN299 GBM cells, and ShRNA-mediated FLNC knocked-down KNS81 and U87MG cells. The gelatin zymography assay was used to estimate matrix metalloproteinase (MMP) 2 activity.

**Results:**

In silico analysis of GBM patient data from TCGA and immunohistochemical analyses of clinical GBM specimens revealed that increased FLNC expression was associated with poor patient prognosis. FLNC overexpression in GBM cell lines was positively correlated with enhanced invasiveness, but not migration, and was accompanied by upregulation of MMP2.

**Conclusions:**

FLNC is a potential therapeutic target and biomarker for GBM progression.

## Background

Glioblastoma multiforme (GBM) is the most aggressive glioma type and accounts for 17% of all primary brain tumours.^1^ The median survival time for GBM patients is 10–11 months with standard treatment including surgical resection and radio-/chemotherapy.^2^ Treatment options that improve survival are limited.^3–5^ GBM progression is associated with several biological and biochemical changes, including cell shape transformation, enhanced mobility and ability to degrade extracellular matrix (ECM), and the emergence of a stem cell phenotype.^6,7^

The cytoskeleton is a complex and dynamic network of protein fibres in eukaryotic cells that determines cell morphology and regulates normal cell activities, including cellular motion, division, and intracellular transport, for instance, in response to external stimuli.^8–11^ These functions depend on structural remodelling, which is mediated by various proteins including filamins (FLNs). FLNs act as a scaffold for over 90 binding partners, including channels, receptors, intracellular signalling molecules, and transcription factors. The three members of the FLN family (FLNA, FLNB, and FLNC) are encoded by different genes and share 60–80% amino acid identity overall and 45% identity in the two hinge regions.^11–13^ FLNs have isoform-specific functions. FLNA is the most abundant and widely distributed member and plays a key regulatory role in apical extrusion^14^; FLNB contributes to angiogenesis by regulating endothelial cell migration^15^; and FLNC is specifically expressed in cardiomyocytes and skeletal myocytes and is involved in the maintenance of structural integrity.^16^ FLN family proteins have been implicated in various cancers.^13^ FLNA overexpression is associated with tumour progression and/or poor prognoses in several cancers including breast cancer,^17–19^ bucca squamous cell carcinoma,^20^ cervical cancer,^21^ prostate cancer^22,23^ and melanoma,^24^ whereas FLNB has been linked to prostate and liver cancer.^23,25^ Although we and others have reported that FLNC is overexpressed in gastric cancer, hepatocellular cancer, and glioma, its precise role in these malignancies is unclear.^26–29^

To address this question in this study, we analysed FLN expression in relation to patient survival using surgically resected GBM specimens as well as by in silico analysis. We also performed gain- and loss-of-function experiments using GBM cell lines to clarify the function of FLNC in GBM progression.

## Materials and methods

### Patients and immunohistochemical analysis of tumour specimens

A total of 90 patients with GBM who underwent surgical treatment at the Department of Neurosurgery, Kagoshima University Hospital from 2008 to 2010 were included in this study. Survival follow-up data were obtained for all patients. The study was approved by the Institutional Review Board of Kagoshima University and was carried out in accordance with the principles outlined in the Declaration of Helsinki. Informed consent was obtained from each patient prior to their inclusion in the study.

Surgically resected tumour samples were fixed in 20% formaldehyde and embedded in paraffin. The tissue blocks were cut into 5-µm sections that were collected onto slides, then deparaffinised and hydrophilised. Antigen retrieval was performed by heating the slides in a microwave in citric acid buffer. Endogenous peroxidase activity was blocked with 3% hydrogen peroxide in methanol followed by incubation in 0.2% TritonX-100 for 10 min. After washing three times with phosphate-buffered saline, the sections were pre-incubated in 5% bovine serum albumin for 30 min to block non-specific reactions, and then incubated overnight at 4 °C with antibodies against FLNA (1:600 dilution), FLNB (1:600 dilution), and FLNC (1:100 dilution). The avidin−biotin complex and immunoperoxidase method (Vectastatin ABC kit; Vector Laboratories, Burlingame, CA, USA) and diaminobenzidine tetra hydrochloride were used to visualise immunoreactivity. Images were captured with a DP73 digital microscope camera (Olympus, Tokyo, Japan). Due to the heterogeneity of GBM tissue samples, we selected four microscopic fields (magnification: ×200) that showed high cellularity and evaluated them with the colour deconvolution method^30^ using ImageJ software (National Institutes of Health, Bethesda, MD, USA).

### Reagents and antibodies

Dulbecco’s modified Eagle’s medium (DMEM) was obtained from Nissui Pharmaceutical (Tokyo, Japan). The following reagents were used: foetal bovine serum (FBS) (Biosera, Kansas City, MO, USA)), l-glutamine (Thermo Fisher Scientific, Waltham, MA, USA), penicillin, sodium pyruvate (Wako Pure Chemical Industries, Osaka, Japan), non-essential amino acids (Thermo Fisher Scientific), G418 (Nacalai Tesque, Kyoto, Japan), and blasticidin (Kaken Pharmaceutical, Kyoto, Japan). Monoclonal antibodies against FLNA (EMD Millipore, Billerica, MA, USA), glyceraldehyde 3-phosphate dehydrogenase (GAPDH; Cell Signalling Technology, Danvers, MA, USA), and FLAG M2 (Sigma-Aldrich, St. Louis, MO, USA); polyclonal antibodies against FLNB (EMD Millipore) and FLNC (Atlas Antibodies, Stockholm, Sweden); and horseradish peroxidase-conjugated anti-rabbit and -mouse IgG (Cell Signalling Technology) were used.

### Cells and cell culture

LN229 (American Type Culture Collection, Manassas, VA, USA), U87MG (HPA Culture Collection, Salisbury, UK), and U251MG and KNS81 (JCRB Cell Bank, Osaka, Japan) human glioblastoma cells were maintained in DMEM supplemented with 10% FBS (except for KNS81 cell cultures, which were supplemented with 5% FBS), 2 mM l-glutamine, 80 U/ml penicillin, and 1 mM sodium pyruvate; and 293FT cells (Thermo Fisher Scientific) were cultured in DMEM containing 10% FBS, 2 mM l-glutamine, 80 U/ml penicillin, 1 mM sodium pyruvate, and 0.1 mM non-essential amino acids. The cell lines were cultured at 37 °C in a humidified atmosphere of 5% CO_2_.

### Plasmid construction and establishment of FLNC-overexpressing cells

A plasmid harbouring the FLNC open reading frame (ORF) (ARi57A02) was provided by RIKEN BioResource Center (Tsukuba, Japan) through the National Bio-Resource, Japan.^31–33^ FLNC and enhanced green fluorescent protein (EGFP) ORFs were ligated into pCDNA3.1(+) (Thermo Fisher Scientific), and pCDNA3.1(+)-FLAG-FLNC and pCDNA3.1(+)-FLAG-EGFP were transfected into LN229 and U251MG cell lines using Lipofectamine 2000 (Thermo Fisher Scientific). Transfected cells were inoculated in a 10-cm^2^ dish (1000 cells/dish) for cloning. After 7 days, colonies derived from a single cell were selected by adding 0.5 mg/ml G418, and FLNC mRNA and protein expression was confirmed.

### Establishment of FLNC knockdown cells

pENTR4-H1 and CS-RfA-EVBsd were obtained from Dr. Hiroyuki Miyoshi at the RIKEN BioResource Center. Short hairpin (sh) RNAs against FLNC (shRNA1 and shRNA2) were designed using siDirect (http://sidirect2.rnai.jp/) and were synthesised by FASMAC (Kanagawa, Japan); the sequences are shown in Supplementary Table S1A. These oligonucleotides along with the negative control scrambled shRNA (SshRNA)^34^ were cloned into the *Bgl*II and *Xba*I restriction sites of the pENTR4-H1 vector. FLNC-shRNA1, FLNC-shRNA2, and SshRNA were separately inserted into the CS-RfA-EVBsd destination vector using LR Clonase II (Thermo Fisher Scientific) according to the manufacturer’s instructions.

293FT cells were seeded in six-well plates and transfected with lentiviral vector plasmid (CS-FLNC-shRNA1-EVBsd, CS-FLNC-shRNA2-EVBSd, or CS-SshRNA-EVBsd) and packaging plasmids (pMDLg/pRRE, pRSV-Rev, and pMD2.G; Addgene, Cambridge, MA, USA) using Lipofectamine 2000 at 37 °C for 72 h. U87MG and KNS81 cells seeded in six-well plates were infected with lentivirus at 37 °C for 48 h. Infectivity was maintained by adding 10 µg/ml blasticidin to the culture medium at every passage.

### Transwell migration and invasion assays

Migration and invasion assays were performed using 8-µm BioCoat 24-well Control Inserts (Corning Inc., Corning, NY, USA) and 8-µm BioCoat 24-well Matrigel Invasion Chamber (Corning Inc.), respectively. A total of 20,000–100,000 cells (depending on the cell line) were seeded in serum-free medium in the top chamber, and the bottom chamber was filled with medium containing 10% FBS. For the U87MG cell line, the chamber membrane was coated with 10 µg/ml fibronectin (Sigma-Aldrich). At 24 h after seeding (48 h for KNS81 cells), the bottom membranes were fixed with 4% paraformaldehyde and stained with haematoxylin. Cells were counted in five random fields under a microscope at ×200 magnification. The number of invading cells was normalised to the number of migrated cells (I/M ratio). To neutralise activated MMP2, 10 nM GM6001 (Cayman chemical, MI, USA) was applied after seeding the cell lines into the Transwell columns.

### RNA isolation and cDNA synthesis

Total RNA from cultured cells was isolated using TRIzol reagent (Thermo Fisher Scientific) and reverse transcribed using the ReverTra Ace kit (Toyobo, Osaka, Japan) according to the manufacturer’s instructions.^35^

### Quantitative real-time (qRT)-PCR

The mRNA expression levels of FLNA, FLNB, and FLNC were determined by qRT-PCR (Step One Plus; Thermo Fisher Scientific) using Go Taq qPCR Master Mix (Promega, Madison, WI, USA) according to the manufacturer’s instructions. Human GAPDH was used for normalisation. Target gene expression was quantified with the comparative cycle threshold method. Forward and reverse primer sequences are shown in Supplementary Table S1B.

### Protein extraction and immunoblotting

Total cell lysate was isolated with radio-immuno-precipitation assay buffer composed of 25 mM Tris-HCl (pH 7.5), 150 mM NaCl, 1% Nonidet P-40, 0.1% sodium dodecyl sulphate (SDS), and 0.5% sodium deoxycholate with proteinase inhibitor cocktail (Nacalai Tesque). Protein concentration was measured using a protein assay kit (Bio-Rad, Hercules, CA, USA). Cell lysates (20 µg protein for FLNA and FLNC and 50 µg for FLNB) were separated by electrophoresis on a 5–20% SDS-polyacrylamide gel (ATTO, Tokyo, Japan) and transferred to a polyvinylidene difluoride membrane (EMD Millipore), which was incubated overnight at 4 °C with anti-FLNA (1:1,000,000 dilution), anti-FLNB (1:1000), anti-FLNC (1:1000), or anti-GAPDH (1:30,000) antibody as previously described.^36^

### Gelatin zymography

The gelatin zymography assay was performed using a commercial kit (Cosmo Bio, Tokyo, Japan) according to the manufacturer’s instructions. Cell culture supernatants were collected and centrifuged at 1500 rpm for 5 min. The cell-free supernatant was mixed with 2× sample buffer and electrophoresed on a precast gel (10% polyacrylamide, 0.1% gelatin) at 4 °C for 1 h. Enzymatic reactions were carried out overnight at 37 °C. Gelatinase activity was visualised using staining solutions and the gel was destained in acetic acid/methanol/dH_2_O (1:3:6).

### In silico analysis of FLNC expression

RNA-seq bam files aligned to human genome (hg38) and survival information of GBM patients in TCGA (The Cancer Genome Atlas) project were downloaded from the Genomic Data Commons (GDC) portal site (https://portal.gdc.cancer.gov). These files were analysed by a series of cufflinks software programs, cuffquant and cuffnorm, to quantify and normalise the expression of the *FLNC* gene. Mutation status of the isocitrate dehydrogenase 1 (*IDH1*) gene in GBM patients was downloaded from cBioPortal (http://www.cbioportal.org/). Patients were divided into two groups by the best cut-off point: high FLNC expression and low FLNC expression, and then, a survival curve was constructed via the Kaplan−Meier method using survfit (R package). *P* values were calculated from the log-rank test using survdiff (R package).

To determine whether FLNC expression in GBM is significantly associated with invasion- and metastasis-related genes, gene set enrichment analysis (GSEA) was carried out with the mRNA expression data from TCGA dataset using software provided by the Broad Institute (http://software.broadinstitute.org/gsea/index.jsp).^37^ We performed GSEA for GO_LAMELLIPODIUM, KEGG_FOCAL_ADHESION, GO_INVADOPODIUM, ALONSO_METASTASIS_UP, CROMER_METASTASIS_UP, CHANDRAN_METASTASIS_UP, and LIAO_METASTASIS gene sets, which represented specific and well-defined biological states or processes and showed coherent expression.

### Statistical analysis

EZR (Saitama Medical Centre, Jichi Medical University)^38^ featuring a graphical user interface for R (The R Foundation for Statistical Computing) was used for all data analysis. Group differences were evaluated with the *χ*^2^ and Student’s *t* tests. Patients were divided into high and low FLNC expression groups based on median FLNA, FLNB, and FLNC expression levels. Kaplan−Meier survival curves were generated by comparing these two groups with the Wilcoxon test. Univariate and multivariate Cox regression analyses were performed. Differences were considered significant at *p* *<* 0.05.

## Results

### High FLNC expression is associated with poor prognosis in GBM patients

**Fig. 1:**
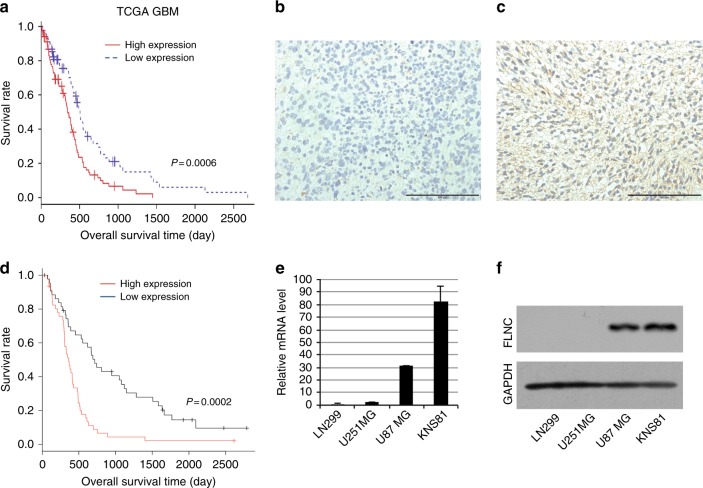
Immunohistochemical detection of FLNC and Kaplan−Meier plot of overall survival in GBM patients. **a** Kaplan−Meier curves showing validation of the prognostic potential of FLNC mRNA expression in TCGA dataset (*N* = 153 high 84 and low 61; *p* = 0.0006). **b**, **c** Representative image of FLNC expression in a GBM specimen; tumours with low (**b**) and high (**c**) expression are shown. Original magnification: ×200; scale bar: 500 μm. **d** Kaplan−Meier curves showing the influence of FLNC expression—with median FLNC level as the cut-off point—on GBM patient outcome in terms of overall survival. Differences were estimated with the log-rank test (*p* = 0.0002). **e**, **f** FLNC mRNA and protein expression levels in GBM cell lines (LN229, U251MG, U87MG, and KNS81) were examined by qRT-PCR and immunoblotting, respectively. GAPDH was used as a loading control. GBM glioblastoma multiforme, TCGA The Cancer Genome Atlas, GAPDH glyceraldehyde 3-phosphate dehydrogenase

Although FLN gene expression is reportedly related to the malignant phenotype of many cancers, it is not well defined in GBM. To investigate whether FLN expression is related to GBM patient outcome, we carried out a Kaplan−Meier analysis of overall survival using TCGA data. High FLNC expression was significantly associated with a poor outcome as compared to low expression (Fig. 1a). However, FLNA and FLNB expression levels were unrelated to prognosis (Supplementary Fig. S1A, B). To determine whether FLNC protein expression is associated with GBM patient prognosis, we examined immunohistochemical, clinical specimens from 90 GBM cases that were divided into two groups based on the median of FLNC expression (Fig. 1b, c). Overall survival was significantly shorter for patients in the high than for those in the low FLNC expression group (*p* < 0.001) (Fig. 1d). Consistent with the analyses of TCGA data, there were no differences in overall survival between high and low FLNA or FLNB expression groups (Supplementary Fig. S2). There were no significant associations between FLNC expression and age, sex, Karnofsky performance status (KPS) score, extent of surgical resection (EOR), and operation time (Supplementary Table S2). We also did not find any significant difference between age and gender in TCGA data for the FLNC high and low expression groups (Supplementary Table S3). Consistent with the analyses of TCGA data, there were no differences in overall survival between high and low FLNA or FLNB expression groups (Supplementary Fig. S2).

We performed a univariate and multivariate Cox proportional hazards analysis to predict outcomes in our GBM patient data. The analysis included clinical features such as age, gender, KPS score, chemotherapy, radiotherapy, and EOR degree in addition to the profiles of the MIB1 percentage and FLNC expression in patient tumour tissues. The analysis showed chemotherapy, radiotherapy, EOR degree and FLNC expression are independent prognostic factors (Table 1). Similar analysis in TCGA data also indicated that FLNC expression in GBM is an independent prognostic factor (Supplementary Table S4).

**Table 1:** Univariate and multivariate analyses in patients with GBM for overall survival (Cox regression analyses)

	Univariate analyses			Multivariate analyses		
	HR	95%CI	*P* value	HR	95%CI	*P* value
Age	1.017	0.998–1.037	0.083	—	—	—
Gender	1.021	0.659–1.581	0.925	—	—	—
KPS score	0.587	0.367–0.938	0.026	0.814	0.493–1.346	0.423
Chemotherapy	0.29	0.146–0.576	<0.001	0.445	0.205–0.967	0.04
Radiotherapy	0.982	0.973–0.991	<0.001	0.9891	0.979–0.999	0.033
EOR degree	0.476	0.308–0.737	<0.001	0.576	0.367–0.904	0.017
MIB1	0.999	0.985–1.014	0.918	—	—	—
FLNC expression	2.497	1.559–4.00	<0.001	2.094	1.308–3.353	0.002

Univariate analyses and multivariate analyses were performed using Ccox regression analyses

*HR* hazard ratio, *CI* confidence interval, *GBM* glioblastoma multiforme, *KPS* Karnofsky performance status, *EOR* extent of surgical resection

*P* < 0.05 was considered statistically significant

### Characterisation of FLNC overexpression and FLNC knockdown cells

**Fig. 2:**
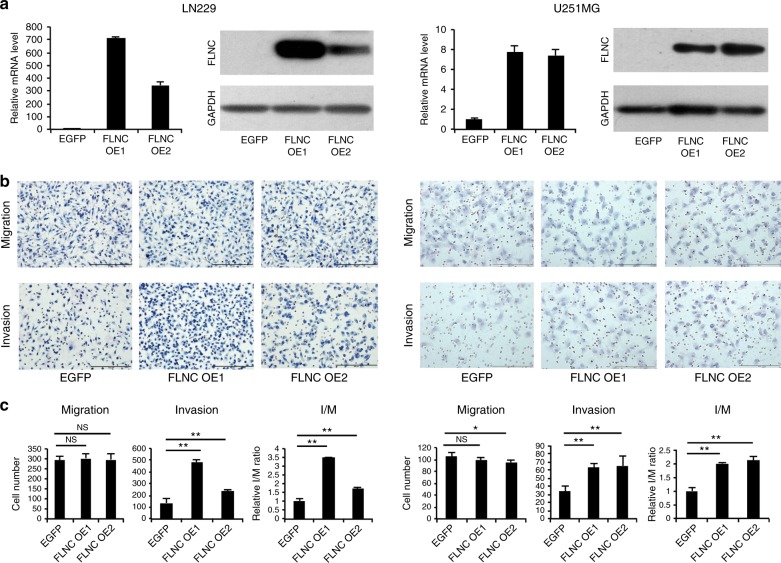
FLNC overexpression enhanced GBM cell invasion. **a** FLNC mRNA and protein levels in control and FLNC-overexpressing (OE) LN229 (left) and U251MG (right) cells, as determined by qRT-PCR and immunoblotting. GAPDH was used as a loading control. **b** Representative images from the Transwell migration and invasion assays of FLNC OE cells. Original magnification: ×200; scale bar: 500 μm. **c** Quantification of control and FLNC OE cell migration and invasion. I/M indicates the invasion/migration ratio. Columns represent total cell number in five independent microscopic fields and bars indicate SD. NS not significant; *P < 0.05; **P < 0.01. GBM glioblastoma multiforme, GAPDH glyceraldehyde 3-phosphate dehydrogenase

**Fig. 3:**
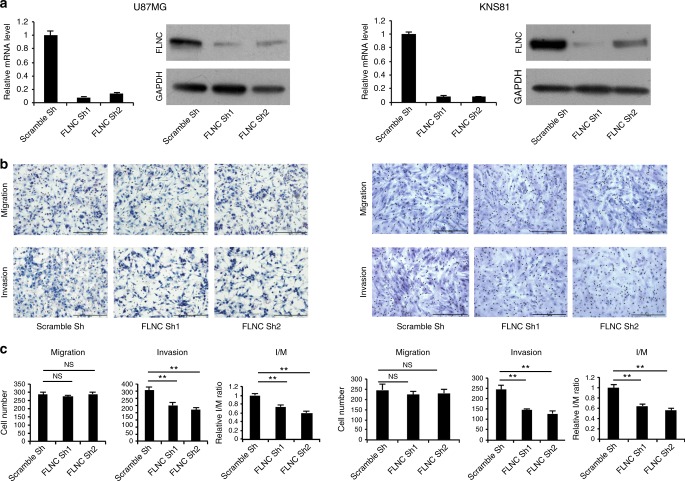
FLNC silencing inhibits invasiveness in GBM cell lines. **a** FLNC mRNA and protein levels of control and U87MG (left) and KNS81 (right) FLNC knockdown (sh) cells, as determined by qRT-PCR and immunoblotting, respectively. GAPDH was used as a loading control. **b** Representative images from the Transwell migration and invasion assays of control and FLNC-depleted cells. Original magnification: ×200; scale bar: 500 μm. **c** Quantification of control and FLNC sh cell migration and invasion. I/M indicates the invasion/migration ratio. Columns represent total cell number in five independent microscopic fields and bars indicate SD. NS not significant; **P < 0.01. GBM glioblastoma multiforme, GAPDH glyceraldehyde 3-phosphate dehydrogenase

We estimated the FLNC expression in a number of GBM cell lines and found that FLNC mRNA and protein levels were much higher in U87MG and KNS81 cells than in LN229 and U251 MG cells (Fig. 1e, f). We therefore established FLNC overexpression cell lines from LN299 and U251MG cells and shRNA-mediated FLNC knockdown cells from U87MG and KNS81 cells. FLNC overexpression or depletion was confirmed by qRT-PCR analysis and western blotting (Figs. 2a and 3a). FLNA or FLNB expression was unaffected by FLNC overexpression and FLNC knockdown in these cells (Supplementary Fig. S3).

### FLNC overexpression and knockdown affect GBM cell invasion but not migration

We evaluated the role of FLNC in GBM cell migration and invasion with the Transwell assay and Transwell Matrigel assay, respectively. FLNC overexpression had no effect on the number of migrated cells but markedly increased the number of invaded cells. This effect of FLNC expression on cell invasion was supported by the increased I/M ratio (Fig. 2b, c). Conversely, FLNC knockdown impaired cell invasion in both U87MG and KNS81 FLNC knockdown cells without altering migration (Fig. 3b, c). Taken together, these results indicate that FLNC promotes GBM cell invasion but has no effect on migration.

### FLNC overexpression induces MMP2 activation

**Fig. 4:**
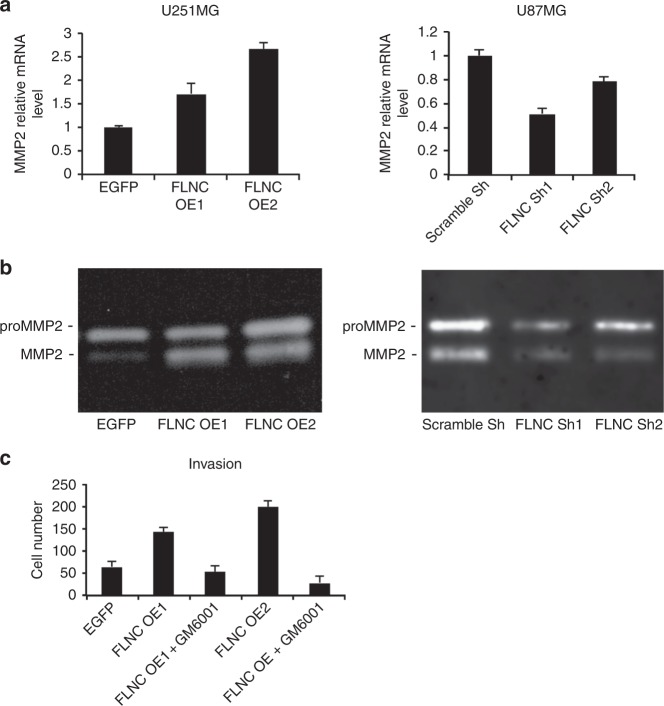
Effect of FLNC signalling on MMP2 activation. **a** MMP2 mRNA expression levels and FLNC were examined by qPCR using cell lysates. **b** U251MG EGFP OE cells and FLNC OE cells as well as U87MG Scramble Sh and FLNC KD cells were incubated in serum-free medium for 48 h, and the conditioned medium was analysed by gelatin zymography assay. The experiment was replicated three times. **c** The graph shows the numbers of U251MG EGFP, FLNC OE1, and FLNC OE2 cells that invaded through the Transwell Matrigel membrane in the presence or absence of GM6001, an MMP2 inhibitor. MMP matrix metalloproteinase

To determine whether FLNC enhances GBM invasion by inducing ECM remodelling, we analysed MMP2 expression by qRT-PCR and examined MMP2 activation by gelatin zymography. FLNC-overexpressing U251MG cells showed increases in MMP2 transcript level, whereas the opposite was observed in FLNC knockdown U87MG cells (Fig. 4a, b). To determine the role of MMP2 in the invasion and migration of FLNC-expressing GBM cells, we used GM6001 an MMP2 inhibitor. The result indicated that invasion but not migration abilities of the cells were depressed by suppression of MMP2 function (Fig. 4c and Supplementary Fig. S4). These data suggest that FLNC enhances the ability of GBM cells to invade the surrounding ECM by inducing MMP2 activation.

### GSEA of the association between lamellipodium-, focal adhesion-, invadopodium-, and metastasis-related gene sets and FLNC expression levels

We carried out GSEA to confirm the relationship between FLNC expression and GBM cell behaviour using TCGA data, including the predefined gene sets GO_LAMELLIPODIUM; KEGG_FOCAL_ADHESION; GO_INVADOPODIUM; ALONSO_METASTASIS_UP; CROMER_METASTASIS_UP; CHANDRAN_METASTASIS_UP; and LIAO_METASTASIS (Supplementary Fig. 5). Consistent with our experimental observations, FLNC upregulation was significantly correlated with the expression of genes related to various aspects of cell motility including lamellipodium, focal adhesion, and invadopodium as well as metastasis.

## Discussion

While other aggressive cancers metastasise via circulatory or lymphatic systems, GBM cells rarely metastasise outside of the brain and instead actively migrate through the perivascular space and spaces between neurons and glial cells that constitute the brain parenchyma and white matter fibre tracts. In order to move through these spaces, glioma cells are presumed to undergo several specific biological changes, including gaining of mobility properties and the ability to degrade ECM, and acquire a stem cell phenotype.^6,39^

In this study, we investigated the role of FLNs in GBM cell invasion. FLNA is thought to be involved in cancer metastasis owing to its involvement in multiple regulatory pathways including hepatocyte growth factor–cMet and Ras signalling.^40,41^ Indeed, FLNA overexpression has been reported to be associated with the invasiveness of cancer. However, recently several researchers have reported tumour suppressive features of FLNA expression in in vitro studies and clinical samples.^19,42–44^ These findings may be consistent with the dual role of FLNA.^45^ We previously reported that elevated FLNC expression in oesophageal squamous cell carcinoma patients is associated with unfavourable prognosis, along with enhanced migration and invasion as well as lymphatic invasion and metastasis. FLNC knockdown in oesophageal squamous cell carcinoma cell lines reduced GTP-Rac1 and GTP-Cdc42 levels, thereby altering cell motility.^28^ However, in GBM cells, FLNC overexpression affected invasion but not migration, and there was no obvious change in Rho activity (Supplementary Fig. S6A). Consistent with these results, FLNC expression in TCGA GBM patient data was unrelated to the GSEA datasets for Rho family proteins by BIOCARTA (Supplementary Fig. S6B). These discrepancies can be explained by the fundamental differences between oesophageal cancer and GBM. GBM cells exhibit sufficient capacity to migrate even in the absence of FLNC expression, whereas oesophageal cancer cells are less motile. The lack of any change in Rho activity suggests that FLNC may activate distinct signalling pathways in GBM and squamous cell cancer cells, although additional studies are needed to evaluate this possibility. FLNC expression was shown to be higher in the glioma than in normal brain tissue and was positively correlated with histological grade of the tumour.^46^ Our data support these findings, although we analysed only high-grade (grade IV) glioma in this study. Parsons and colleagues demonstrated that overall survival in IDH1-mutant GBM was more than threefold higher than that in IDH1 wild-type GBM, and genome wide-analysis has revealed that IDH1 is mutated in approximately 12% of GBM cases.^47^ Excluding the patients carrying IDH mutant GBM from analysis did not change the results of the prognosis analyses (Supplementary Fig. S1 and S2).

FLNC promotes prostate cancer progression by enhancing the migratory capacity of tumour cells.^27^ FLNC was upregulated in hepatocellular carcinoma cells as compared to normal liver cells, and its overexpression induced cell migration; additionally, FLNC knockdown suppressed cell proliferation and migration while enhancing apoptosis, whereas FLNC overexpression enhanced cancer progression and metastasis both in vitro and in vivo.^26,29^ High FLNC expression was also related to poor prognosis in patients. These reports provide evidence that FLNC is related to the aggressiveness of cancers. However, FLNC has also been linked to favourable outcome in gastric cancer; it was shown to be downregulated in gastric cancer cell lines as compared to normal cells and its silencing induced MMP2 activation in vitro.^48^ Thus, FLNC may have opposing functions in cancer similar to what has been reported for FLNA.^40,42^

FLNC is an actin cross-linking cytoskeletal protein that contributes to the regulation of cell morphology, which can facilitate the metastasis of tumour cells. The ability to remodel the ECM is important for tumour cell invasion and dissemination; indeed, the metastatic potential of GBM cells can be enhanced by increasing their motility and/or ability to degrade ECM.^6,7,49^ Our GSEA of TCGA data revealed a significant enrichment of gene signatures associated with lamellipodia, focal adhesion, and invadopodia and also metastatic features, suggesting that FLNC promotes the malignant characteristics of GBM (Supplementary Fig. S5).

The process of ECM degradation is largely attributable to the activation of MMPs, which are a family of structurally related zinc-dependent endopeptidases that are expressed at high levels in the tumour microenvironment and can degrade various ECM components, thereby stimulating cancer cell invasion and metastasis. As such, increased MMP levels in cancer patients are often associated with poor prognosis.^50^ Glioma cells express various MMPs, with MMP2 reported to degrade ECM components most effectively.^49,51^ Our results showed a positive correlation between FLNC level and MMP2 expression and activation, suggesting that FLNC promotes GBM metastasis by stimulating the degradation of the ECM. Plasticity of expression and modification in cytoskeleton-related proteins including FLNC is thought to be essential for remodelling of cellular cytoskeleton in invasion and migration of cells. It is necessary to study about these regulation of FLNC to elucidate its precise function in tumour progression.

In summary, our findings show that high FLNC expression in GBM patients is an independent predictor of unfavourable prognosis and that FLNC increases the invasive potential of GBM via regulation of MMP2 expression and activity. Thus, FLNC is a useful biomarker and a promising therapeutic target in GBM. Identification of FLNC-interacting proteins and downstream signalling pathways will be critical for elucidating its function in future studies and in in vivo experiments.

## Supplementary information

Supplementary Table 1

Supplementary Table 2

Supplementary Table 3

Supplementary Table 4

Supplementary Figure S1

Supplementary Figure S2

Supplementary Figure S3

Supplementary Figure S4

Supplementary Figure S5

Supplementary Figure S6

Supplementary Figure Legends

## Data Availability

The datasets generated and analysed during the current study are not publicly available but available from the corresponding author on reasonable request.
