# Supplementary figures and images for: High filamin-C expression predicts enhanced invasiveness and poor outcome in glioblastoma multiforme
Source: Br J Cancer. 2019 Mar 14;120(8):819–26. doi: 10.1038/s41416-019-0413-x (PMC6474268; doi:10.1038/s41416-019-0413-x)

A

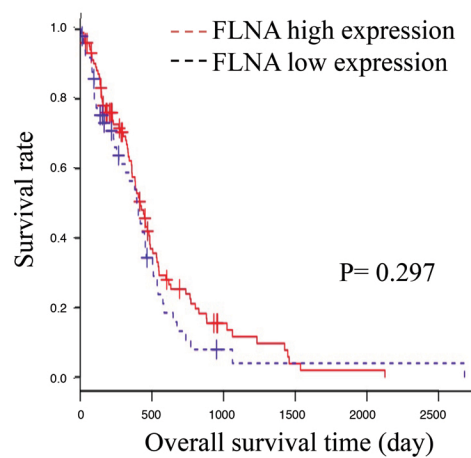

B

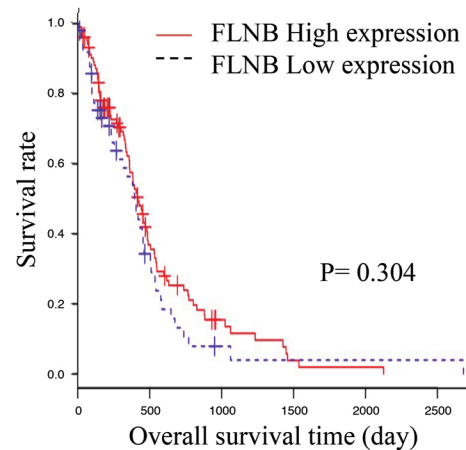

C

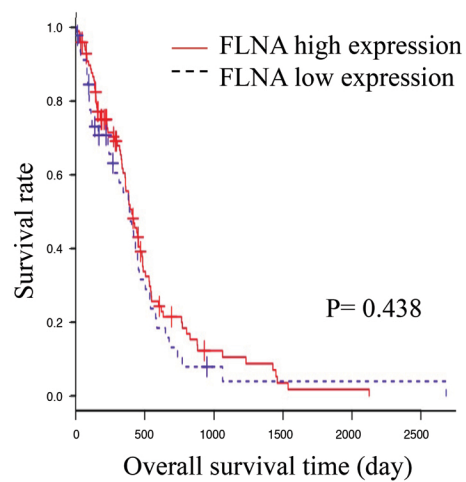

D

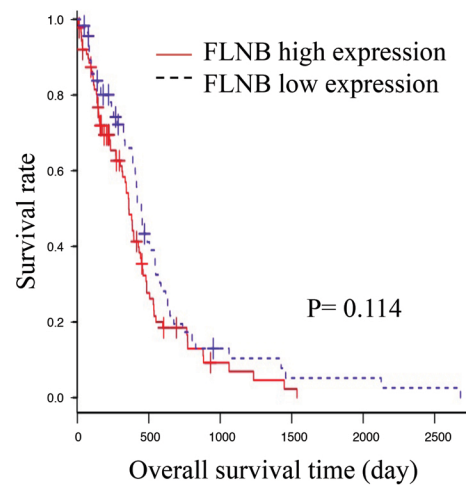

E

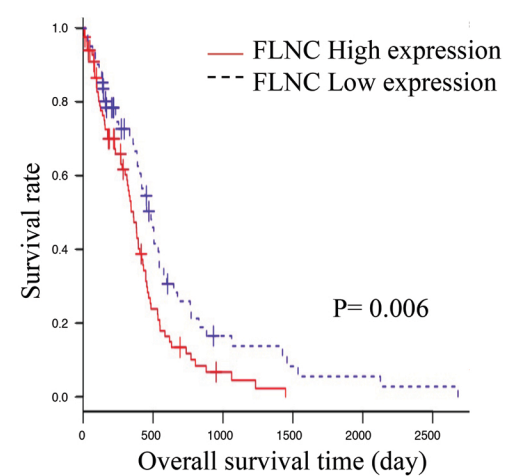

Supplement: Supplementary file 5 — Supplementary Figure S1 [file 41416_2019_413_MOESM5_ESM.pdf]

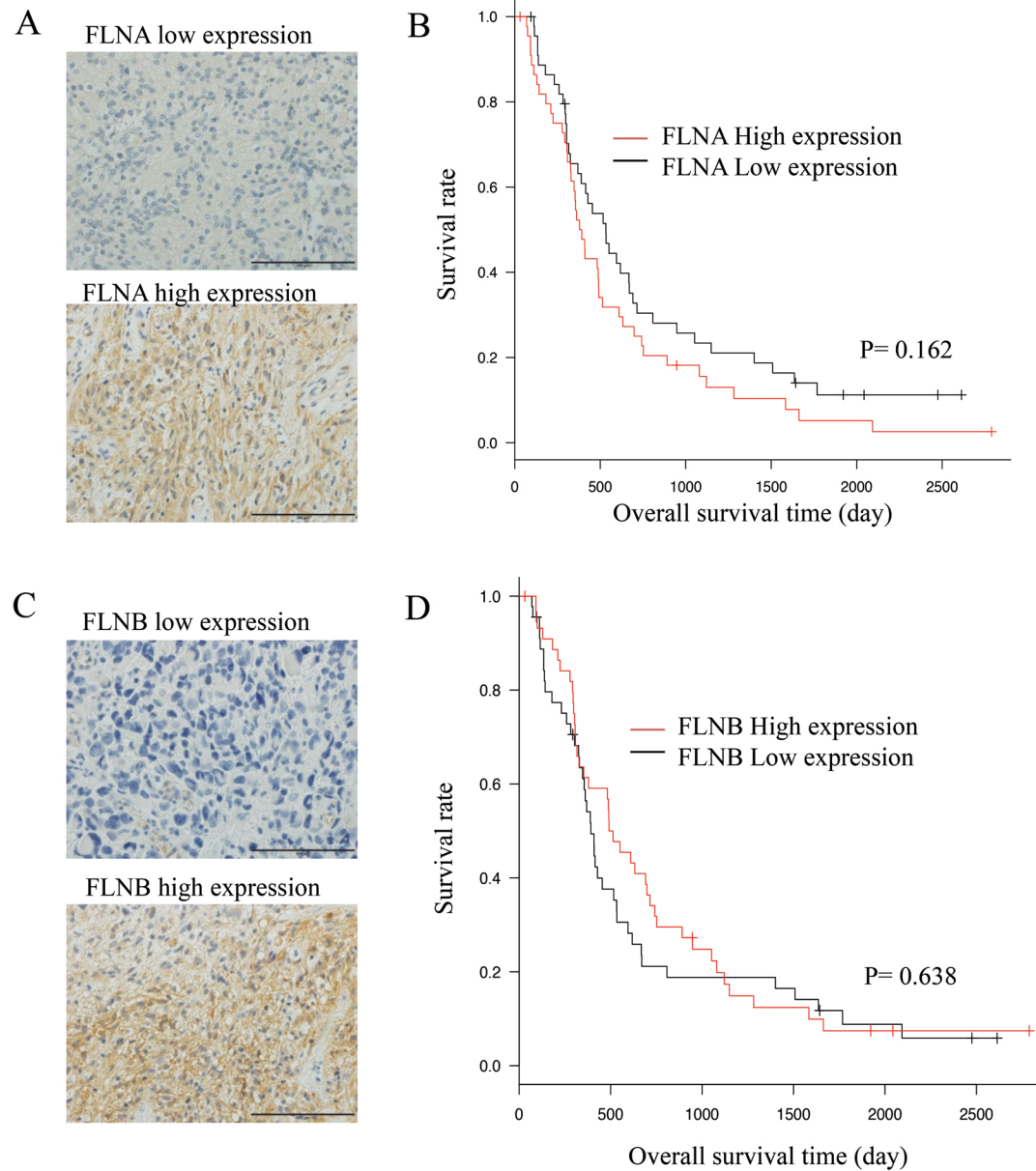

Supplementary Figure S2

Supplement: Supplementary file 6 — Supplementary Figure S2 [file 41416_2019_413_MOESM6_ESM.pdf]

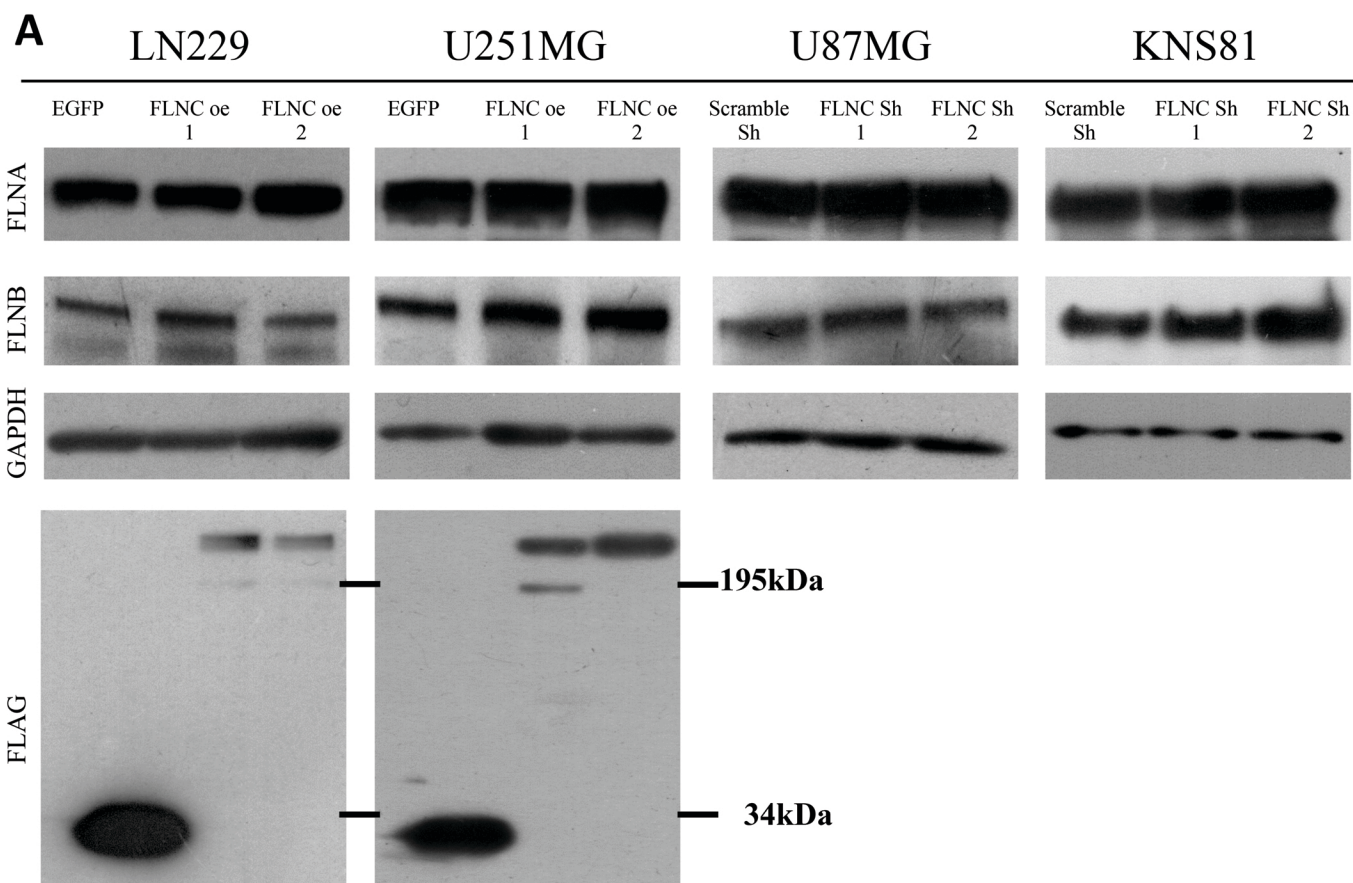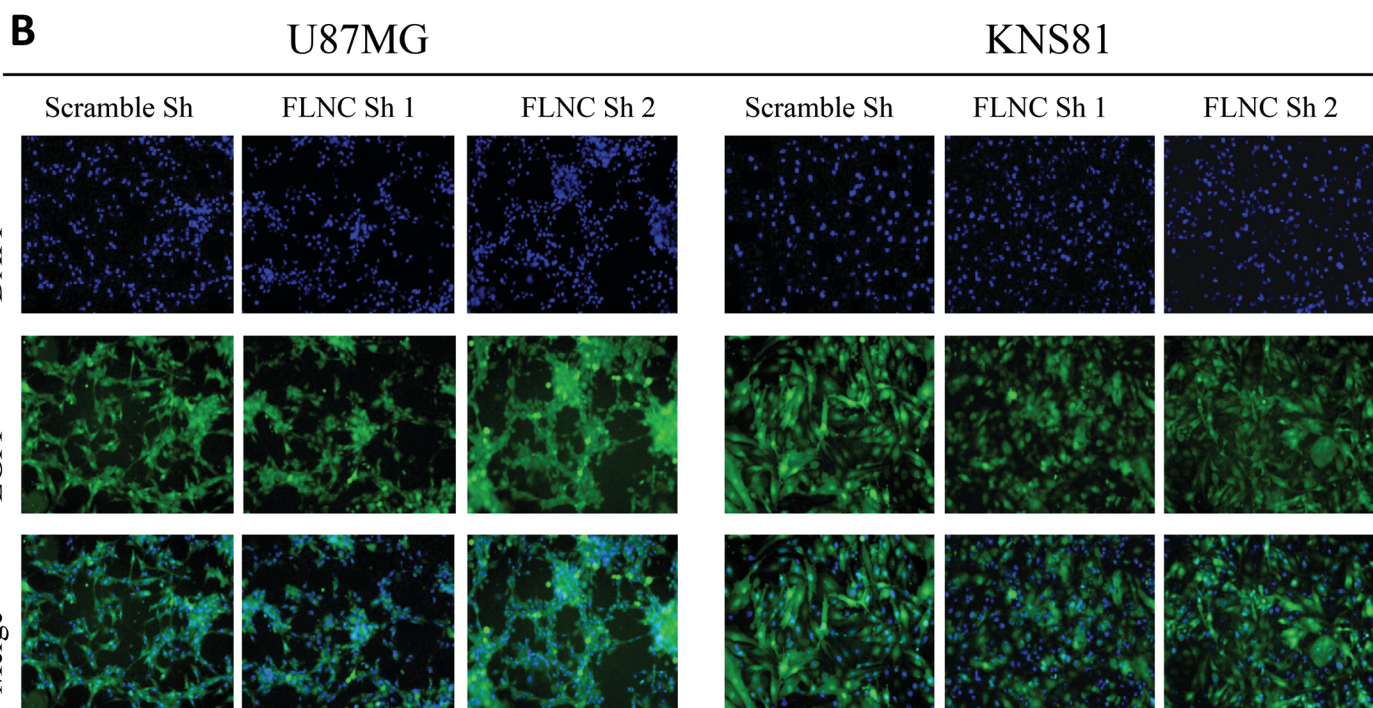

Supplement: Supplementary file 7 — Supplementary Figure S3 [file 41416_2019_413_MOESM7_ESM.pdf]

A

## migration

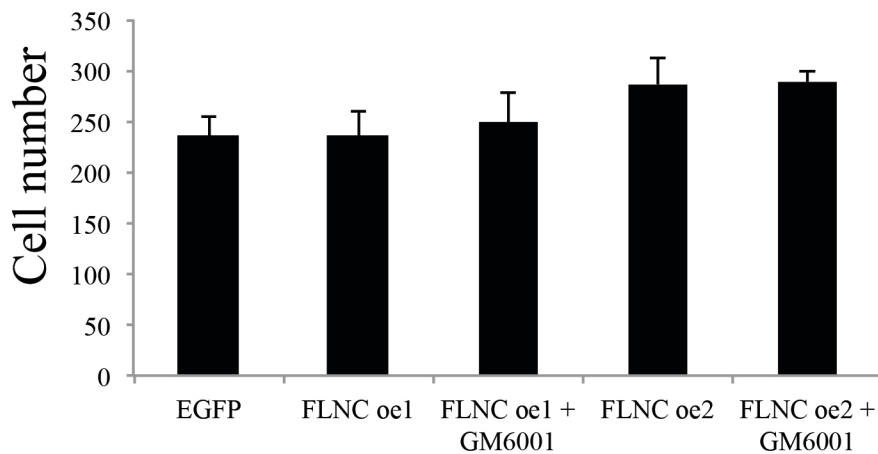

B

## I/M

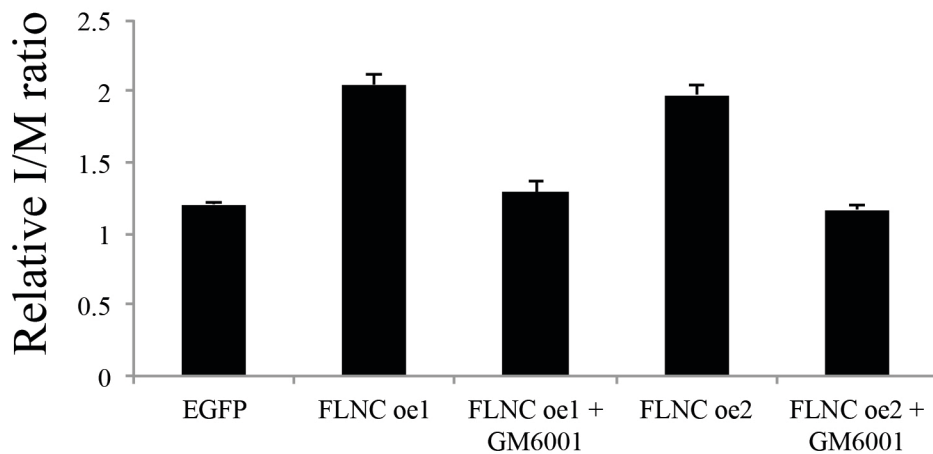

Supplement: Supplementary file 8 — Supplementary Figure S4 [file 41416_2019_413_MOESM8_ESM.pdf]

A

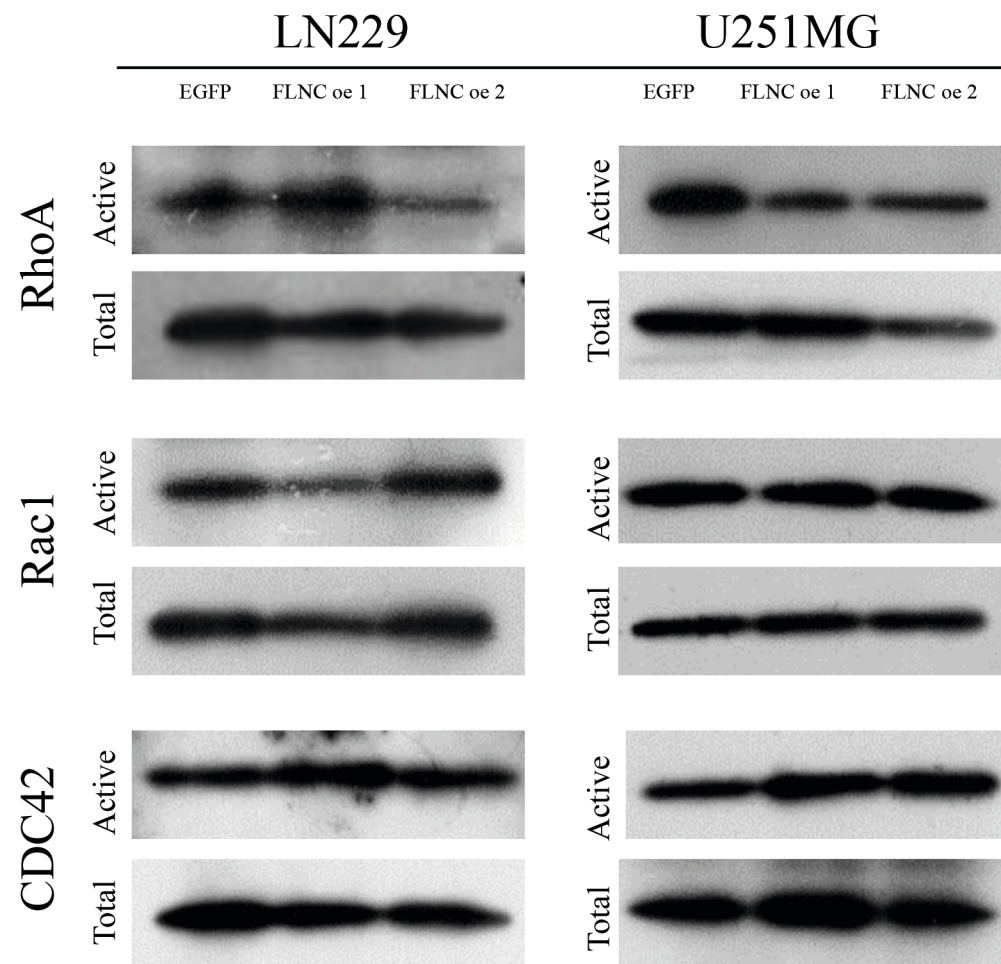

B

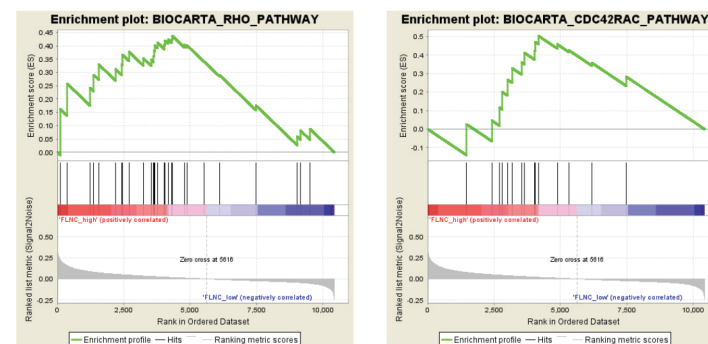

|     |          |     |          |
|-----|----------|-----|----------|
| ES  | 0.437231 | ES  | 0.426364 |
| NES | 1.410995 | NES | 1.101311 |
| P   | 0.10338  | P   | 0.319923 |
| FDR | 0.117838 | FDR | 0.382936 |

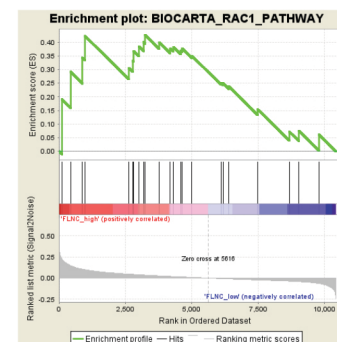

|     |          |
|-----|----------|
| ES  | 0.503481 |
| NES | 1.312286 |
| P   | 0.176699 |
| FDR | 0.176127 |

Supplement: Supplementary file 10 — Supplementary Figure S6 [file 41416_2019_413_MOESM10_ESM.pdf]
