# Supplementary material for: High filamin-C expression predicts enhanced invasiveness and poor outcome in glioblastoma multiforme
Source: Br J Cancer. 2019 Mar 14;120(8):819–26. doi: 10.1038/s41416-019-0413-x (PMC6474268; doi:10.1038/s41416-019-0413-x)
Supplement: Supplementary file 9 — Supplementary Figure S5 [file 41416_2019_413_MOESM9_ESM.pdf]

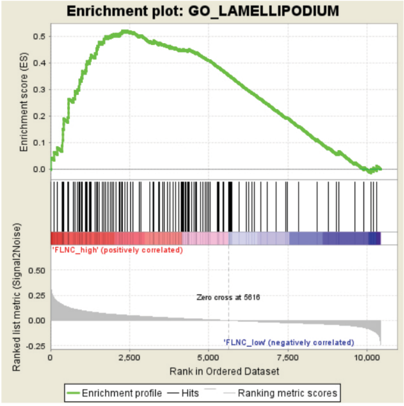

ES	0.519854
NES	1.676679
P	0.002045
FDR	0.053221

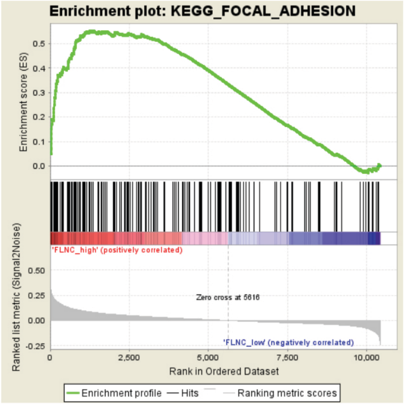

ES	0.552635
NES	1.599389
P	0.018182
FDR	0.090334

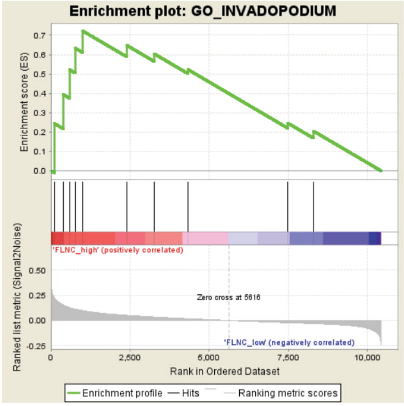

ES	0.722897
NES	1.448991
P	0.060362
FDR	0.060362

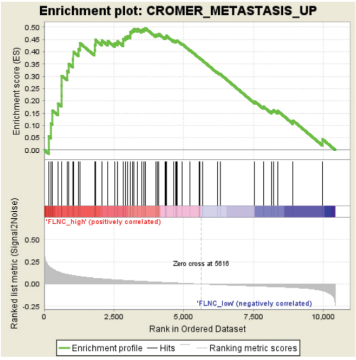

ES	0.495688
NES	1.57787
P	0.026369
FDR	0.088241

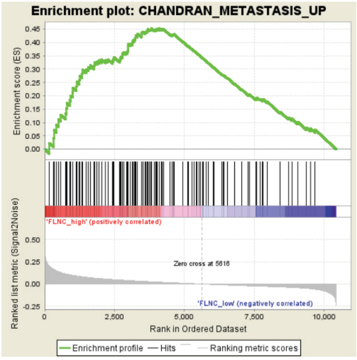

ES	0.452944
NES	1.52185
P	0.028
FDR	0.085388

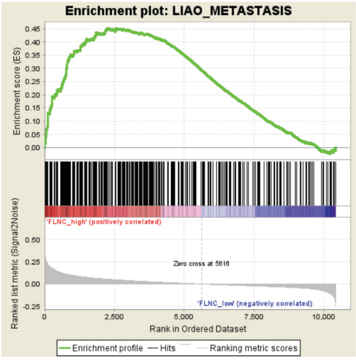

ES	0.452206
NES	1.492587
P	0.014
FDR	0.084202

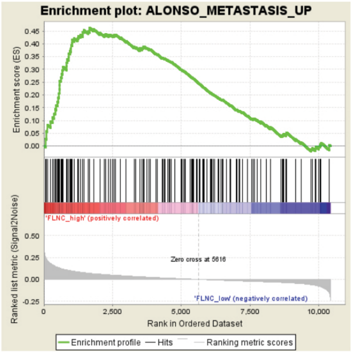

ES	0.462476
NES	1.55828
P	0.02268
FDR	0.084284

Supplementary Figure S5
