# Supplementary material for: High filamin-C expression predicts enhanced invasiveness and poor outcome in glioblastoma multiforme
Source: Br J Cancer. 2019 Mar 14;120(8):819–26. doi: 10.1038/s41416-019-0413-x (PMC6474268; doi:10.1038/s41416-019-0413-x)
Supplement: Supplementary file 11 — Supplementary Figure Legends [file 41416_2019_413_MOESM11_ESM.docx]

**Supplementary Figure S1.** **(A, B)** Kaplan-Meier analyses of the associations between FLNA or FLNB mRNA expression and overall survival of GBM patients in TCGA data set (*N* = 153). **(C, D, E)** Kaplan-Meier analyses of the associations between FLNA, FLNB, or FLNC mRNA expression and overall survival of the GBM patients carrying wild-type IDH1 in TCGA data set (*N* = 146).

**Supplementary Figure S2. (A, C)** Representative images of low and high FLNA and FLNB expression in GBM specimens. Original magnification: 200×; scale bar: 500 μm. **(B, D)** Kaplan-Meier curves showing the influence of FLNA and FLNB levels ---with the median values as the cutoff points---on overall survival of GBM patients. Differences were estimated with the log-rank test.

**Supplementary Figure S3.** (A) FLNA, FLNB, and FLAG protein expression levels as determined by immunoblotting. GAPDH was used as a loading control. FLNA and FLNB expression was unaltered in FLNC-overexpressing (OE) and FLNC knockdown (sh) cells. **(B)** Infection efficiency of shRNA lentiviral vectors in GBM cell lines (U87MG and KNS81), as confirmed by visualising EGFP expression on a confocal microscope. Nuclei were stained with DAPI. More than 90% of cells were infected with the shRNA lentivirus.

**Supplementary Figure S4.**

**(A)** The graph indicates the number of U251MG cells (EGFP-expressing cells and FLNC-overexpressing cells) that migrated through the Transwell membrane in the presence and absence of MMP2 inhibitor, GM6001. **(B)** Invasion/migration ratio showing that the MMP2 inhibitor, GM6001, significantly inhibited the invasive ability of FLNC-overexpressing U251MG cells.

**Supplementary Figure S5.** Identification of enriched gene sets correlated with FLNC expression.

GSEA of FLNC mRNA expression levels from TCGA GBM dataset. Analyses were performed for GO_LAMELLIPODIUM; KEGG_FOCAL_ADHESION; GO_INVADOPODIUM; ALONSO_METASTASIS_UP; CROMER_METASTASIS_UP; CHANDRAN_METASTASIS_UP; and LIAO_METASTASIS gene sets. ES: enrichment score; NES: normalised enrichment score; *P*: nominal P value; FDR: false discovery rate.

**Supplementary Figure S6. (A)** Rho GTPase activity in total lysates was evaluated by a pull-down assay. No changes in GTP-RhoA, GTP-Rac1, and GTP-Cdc42 were observed upon FLNC overexpression (OE). **(B)** GSEA of FLNC mRNA levels from TCGA GBM data using BIOCARTA Rho-family pathway gene sets.
